# Supplementary material for: SARS-CoV-2 inhibits induction of the MHC class I pathway by targeting the STAT1-IRF1-NLRC5 axis
Source: Nat Commun. 2021 Nov 15;12:6602. doi: 10.1038/s41467-021-26910-8 (PMC8594428; doi:10.1038/s41467-021-26910-8)
Supplement: Supplementary file 1 — Supplementary information [file 41467_2021_26910_MOESM1_ESM.pdf]

Supplementary Information

SARS-CoV-2 Inhibits Induction of the MHC Class I Pathway by Targeting the STAT1-IRF1-NLRC5 Axis

Authors

Ji-Seung Yoo[1], Michihito Sasaki[2], Steven X Cho[1], Yusuke Kasuga[1], Baohui Zhu[1], Ryota Ouda[1], Yasuko Orba[2,3], Paul de Figueiredo[4,5], Hirofumi Sawa[2,3,6], and Koichi S Kobayashi[1,5 *]

Affiliations

[1] Department of Immunology, Hokkaido University Graduate School of Medicine, Sapporo 060-8638, Japan.

[2] Division of Molecular Pathobiology, International Institute for Zoonosis Control, Hokkaido University, Sapporo 001-0020, Japan.

[3] International Collaboration Unit, International Institute for Zoonosis Control, Hokkaido University, Sapporo 001-0020, Japan.

[4] Department of Veterinary Pathobiology, Texas A&M University, College Station, TX 77843, USA.

[5] Department of Microbial Pathogenesis and Immunology, Texas A&M Health Science Center, Bryan, TX 77807, USA.

[6] One Health Research Center, Hokkaido University, Sapporo 001-0020, Japan.

* Corresponding author

Supplementary Fig. 1.

a

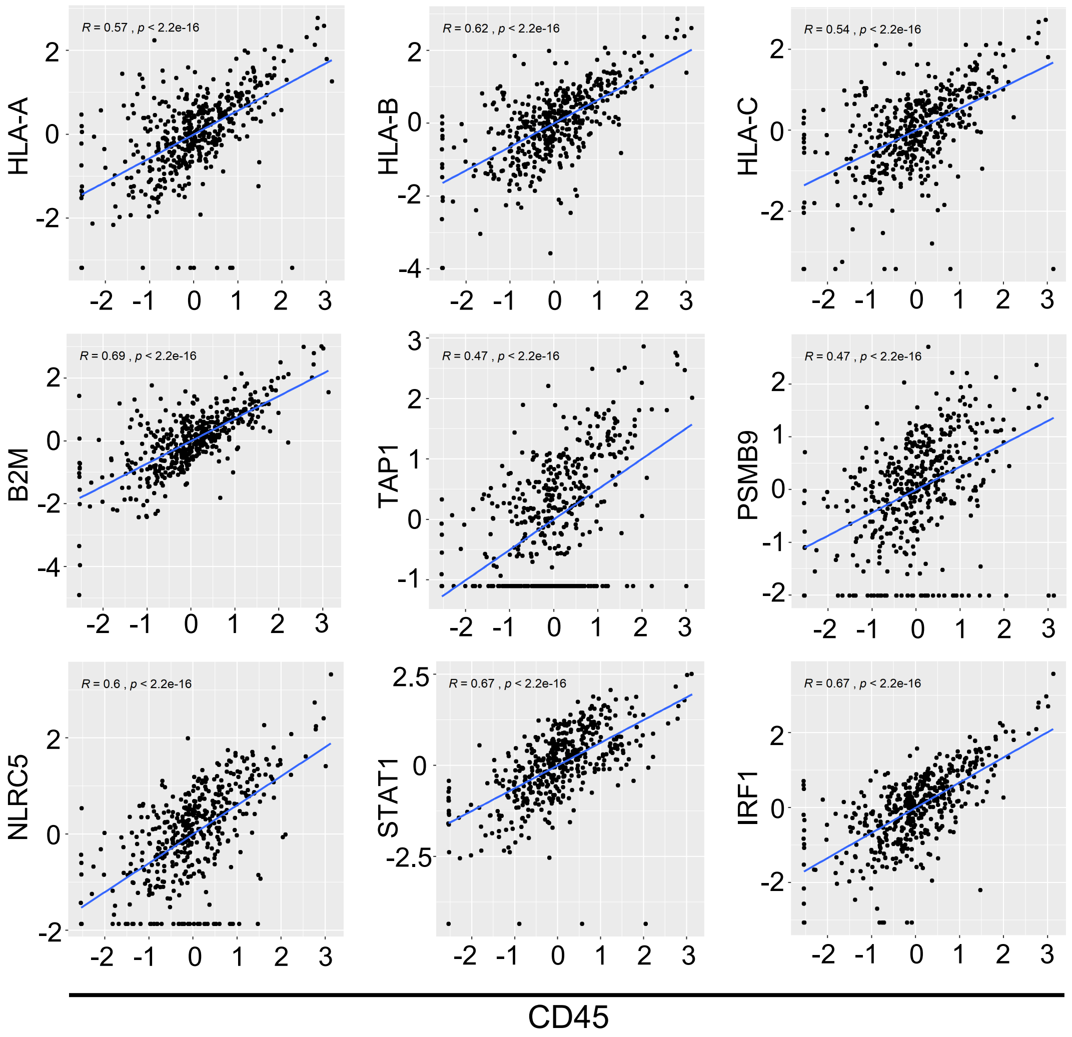

b

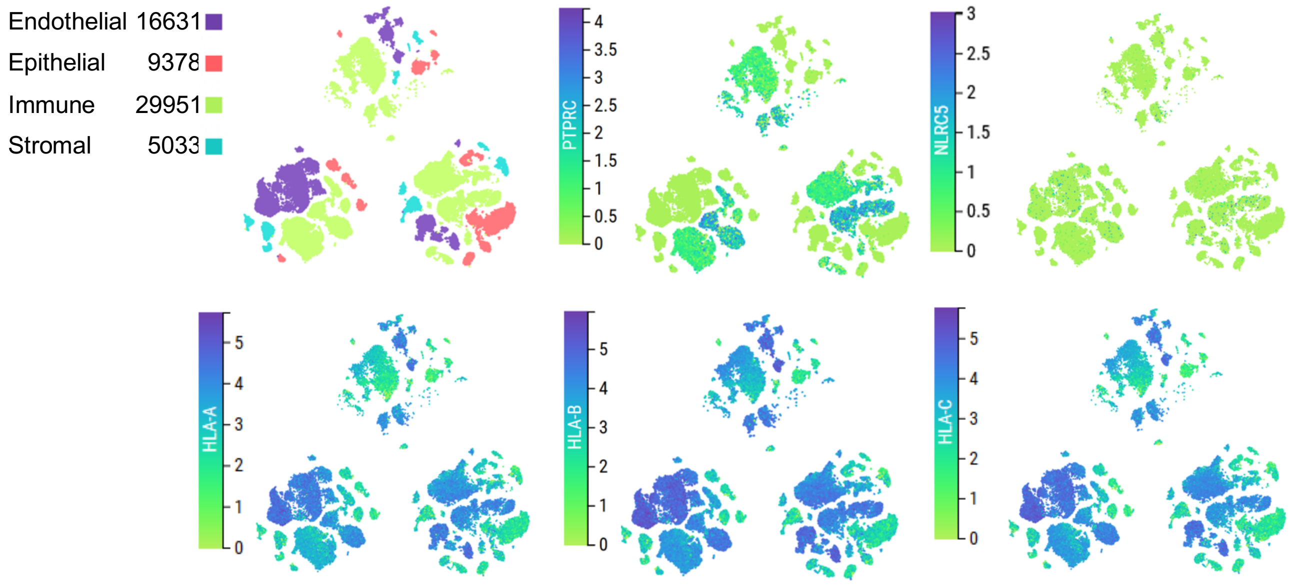

Supplementary Fig. 1. Immune cells are the predominant source of MHC class I gene expression in airway. **a** Scatterplots for the expression of *PTPRC* (CD45) and the indicated MHC class I-related genes from nasopharyngeal swabs of SARS-CoV-2 negative (n=54) and positive (n=413) patients shown in Fig. 1A. Each datapoint represents z-score transformed log2 normalized count for each patient. Spearman's correlation coefficient (*R*) and associated two-sided p-values are indicated. **b** Cellxgene derived (see **Methods**) UMAP representations of single-cell transcriptomes from normal human lung tissue (n=3) annotated by cell type compartments and gene expression of *PTPRC*, *NLRC5*, or MHC class I genes, *HLA-A*, *HLA-B*, and *HLA-C*.

Supplementary Fig. 2.

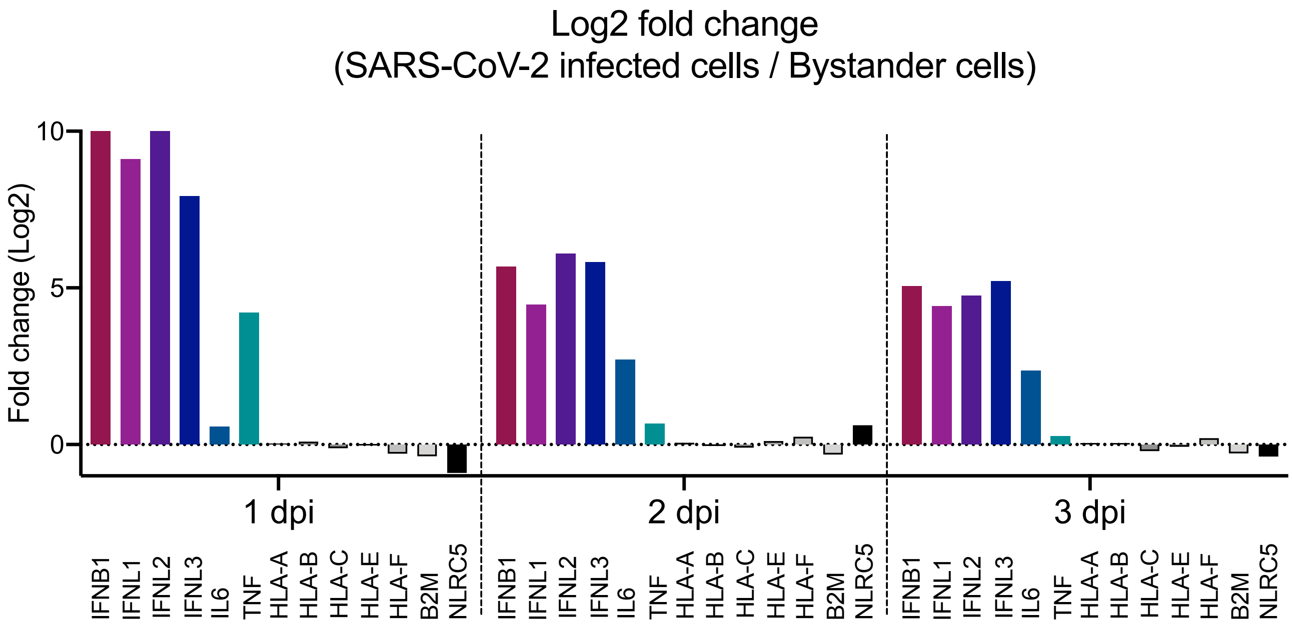

Supplementary Fig. 2. Suppressed upregulation of the MHC class I pathway in SARS-CoV-2 infected ciliated cells Single-cell RNA-seq of SARS-CoV-2 infected primary human bronchial epithelial cells over 1-3 days post-infection (dpi) time course was performed and a comparison of log2 fold change in the indicated gene expression between infected or non-infected bystander ciliated cells was assessed. Data assessment with the provided S2 Data published by Ravindra et al is described in **Methods**.

Supplementary Fig. 3.

a

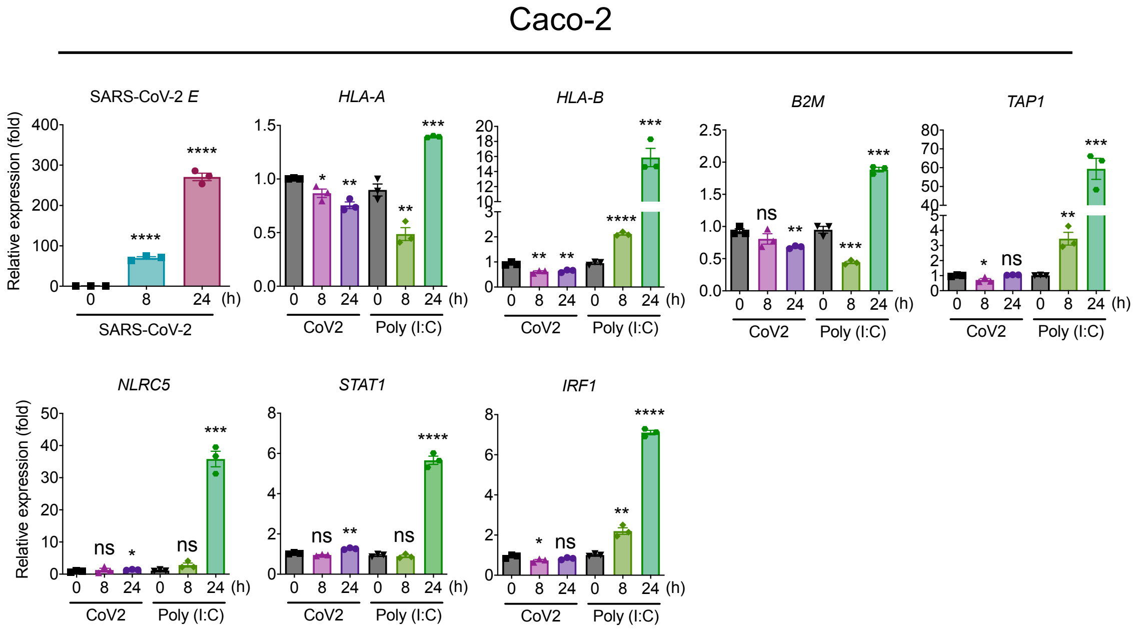

b

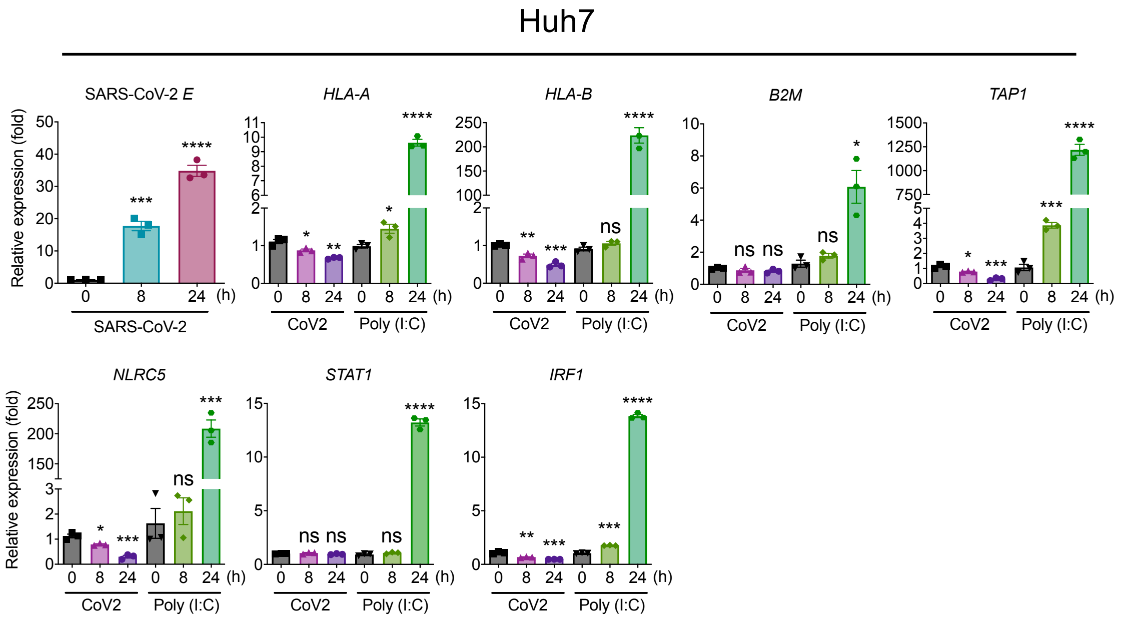

Supplementary Fig. 3. SARS-CoV-2 inhibits the upregulation of genes in the MHC class I pathway in the colon and liver carcinoma epithelial cells. a and **b** Expression of the MHC class I-related genes by SARS-CoV-2 infection (multiplicity of infection (moi) 3) or poly(I:C) transfection (200 ng) for the indicated time in Caco-2 (**a**) or Huh7 cells (**b**). The results are from 3 independent experiments. Two-tailed unpaired *t*-test, *, $P<0.05$; **, $P<0.01$; ***, $P<0.001$; ****, $P<0.0001$; ns, not significant. The error bars represent mean values ± SD. The exact *p*-values are available in the Source Data.

Supplementary Fig. 4.

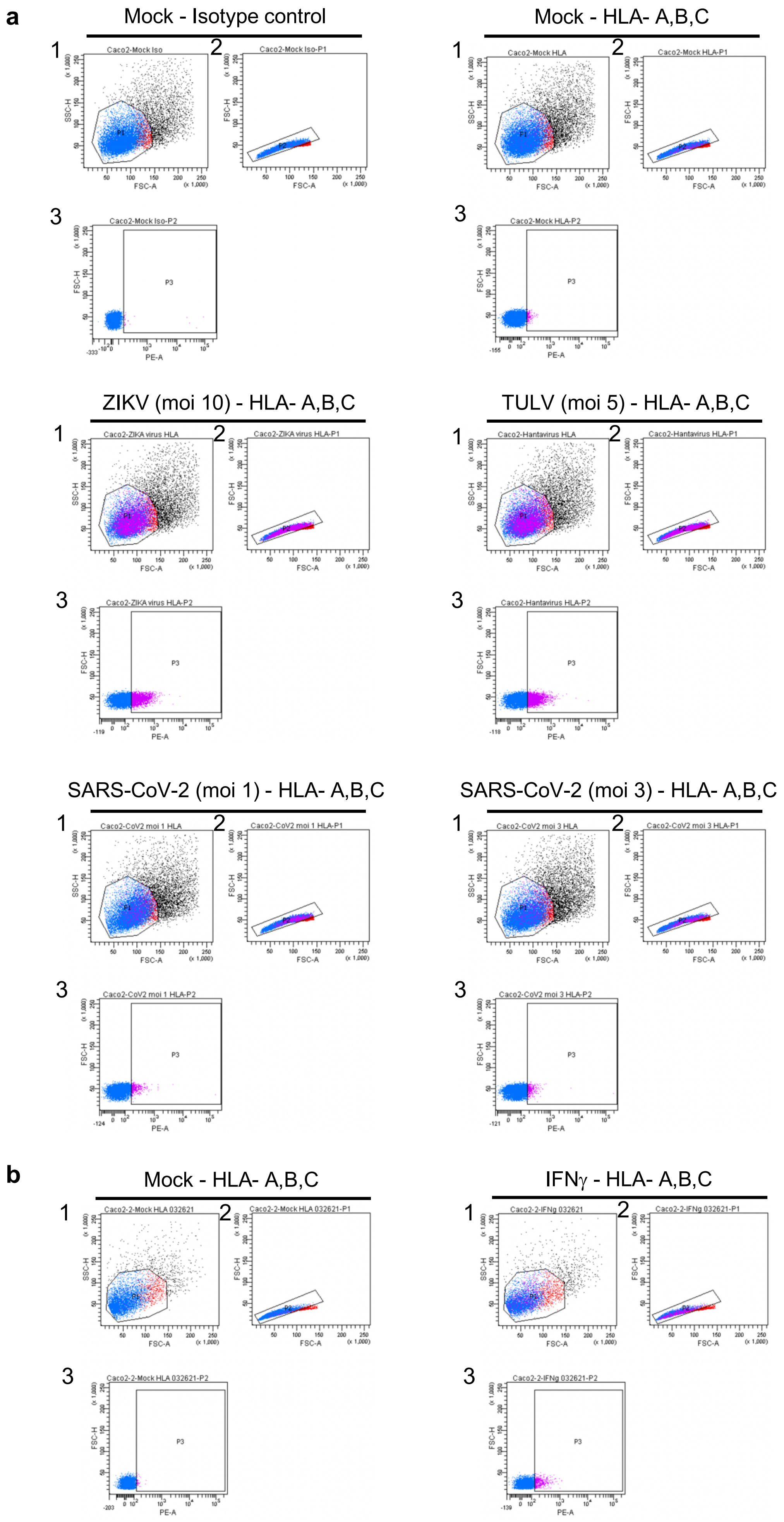

Supplementary Fig. 4. Suppressed upregulation of the surface expression of the MHC class I proteins by SARS-CoV-2 in Caco-2 cells The gating strategy for detection of HLA- A,B,C in Caco-2 cells corresponding to Fig. 2c. (**a**) Surface expression of MHC class I upon mock, ZIKV, TULV, or SARS-CoV-2 infection with indicated moi for 48 h. (**b**) Surface expression of MHC class I by either mock or IFNγ stimulation for 48h. The gating orders are indicated by numbers. Both isotype control and anti-HLA- A,B,C antibodies are conjugated with PE.

Supplementary Fig. 5.

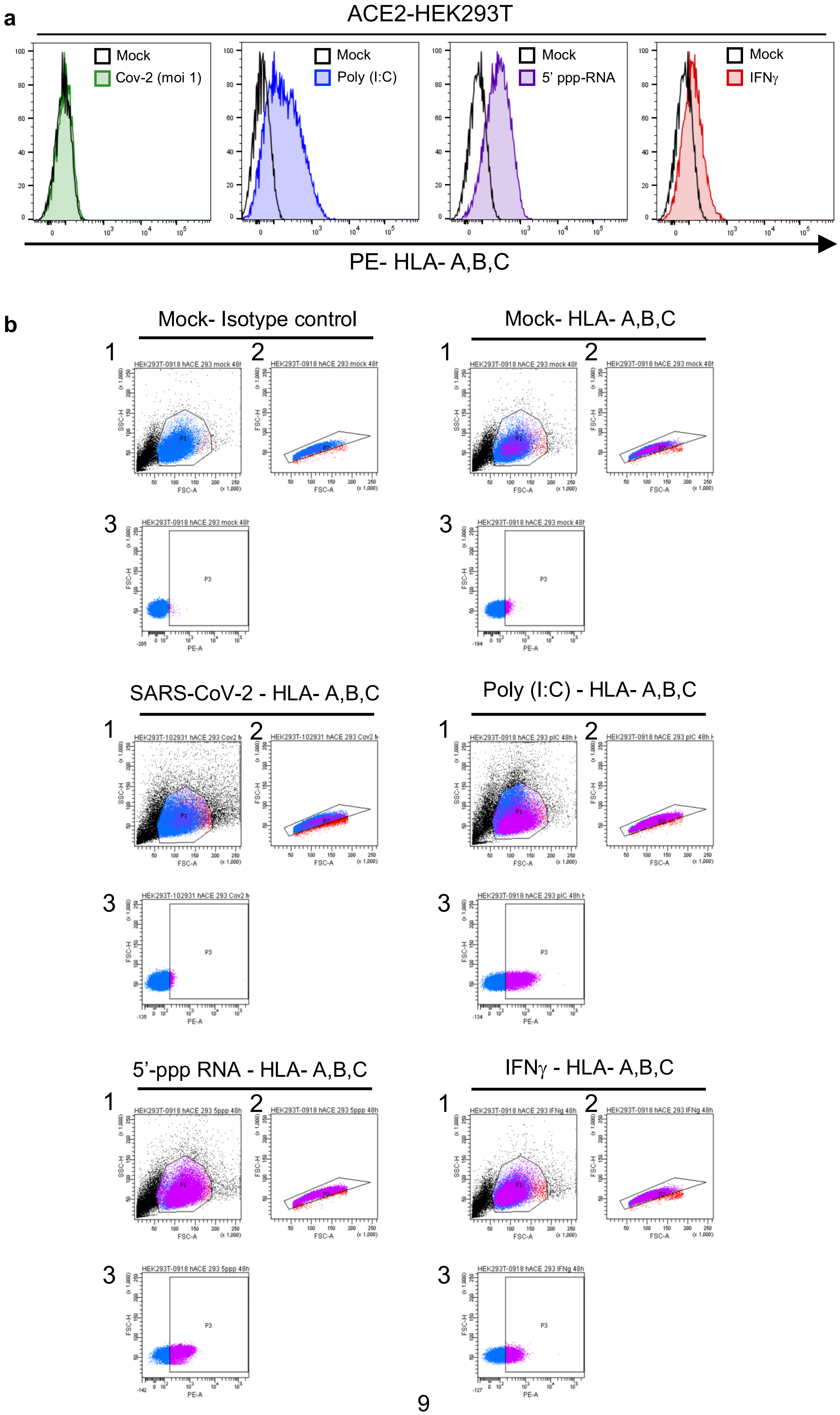

Supplementary Fig. 5. SARS-CoV-2 inhibits upregulation of the surface expression of the MHC class I proteins (**a**) Surface expression of MHC class I upon SARS-CoV-2 infection with indicated moi for 48 h in human ACE2-expressing HEK293T. Stimulation with poly(I:C) (200 ng), 5'ppp RNA (200 ng), or IFNγ (100 U/ml) for 48 h were used as positive controls. (**b**) The gating strategy for detection of the HLA- A,B,C surface expression in ACE2-HEK293T cells. The gating orders are indicated by numbers. Both isotype control and anti-HLA- A,B,C antibodies are conjugated with PE.

Supplementary Fig. 6.

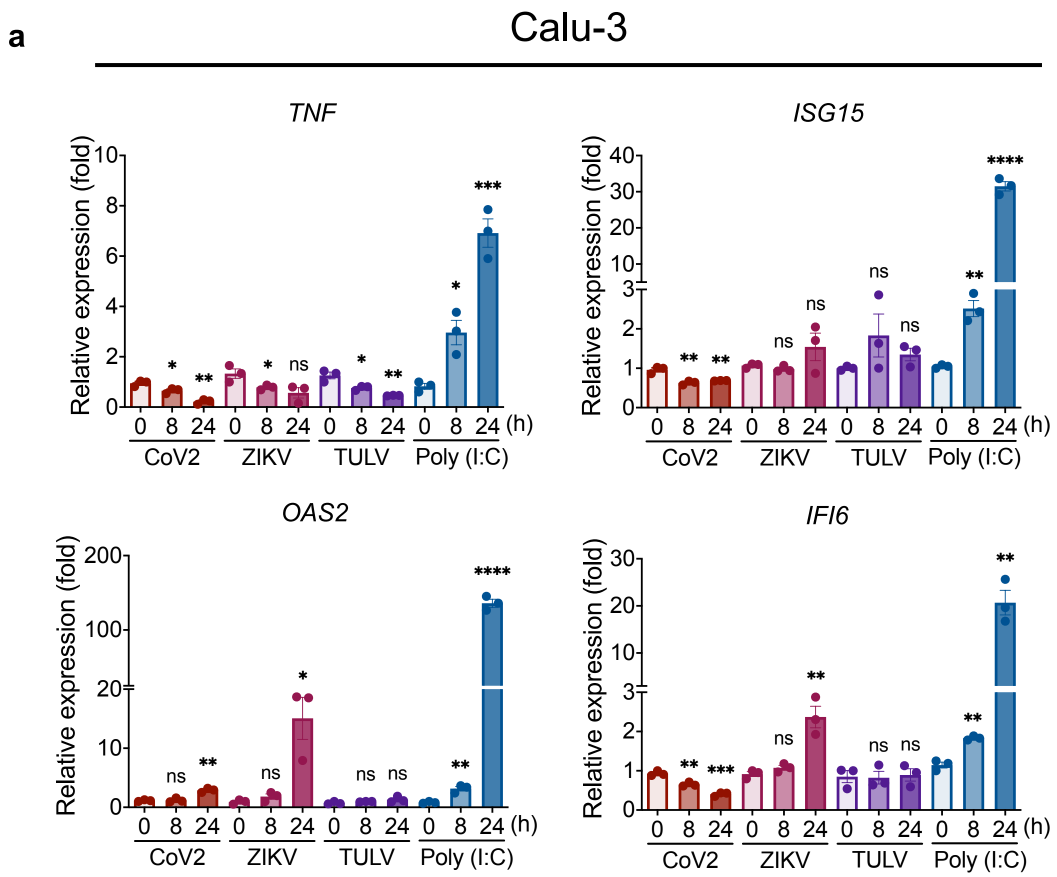

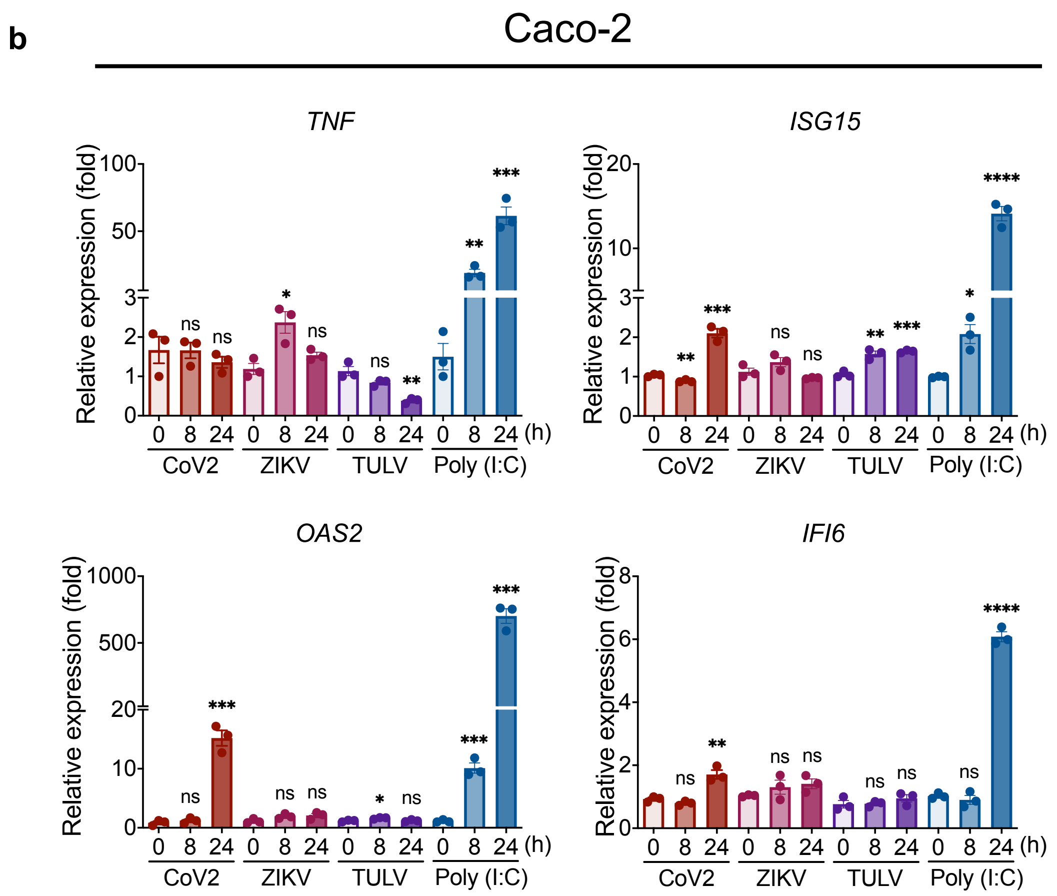

Supplementary Fig. 6. Inhibition of innate immune responses by SARS-CoV-2, Zika virus, and Tula virus in the lung and colon epithelial carcinoma cell lines

Quantitative real-time PCR analysis of the indicated gene expression level in SARS-CoV-2 (moi 3), ZIKV (moi 10), TULV (moi 5)-infected, or poly(I:C) (200 ng)-transfected Calu-3 (**a**) or Caco-2 (**b**) cells for the indicated time. The results are from 3 independent experiments. Two-tailed unpaired *t*-test, *, $P<0.05$; **, $P<0.01$; ***, $P<0.001$; ****, $P<0.0001$; ns, not significant. The error bars depict mean values ± SD. The exact *p*-values are available in the Source Data.

Supplementary Fig. 7.

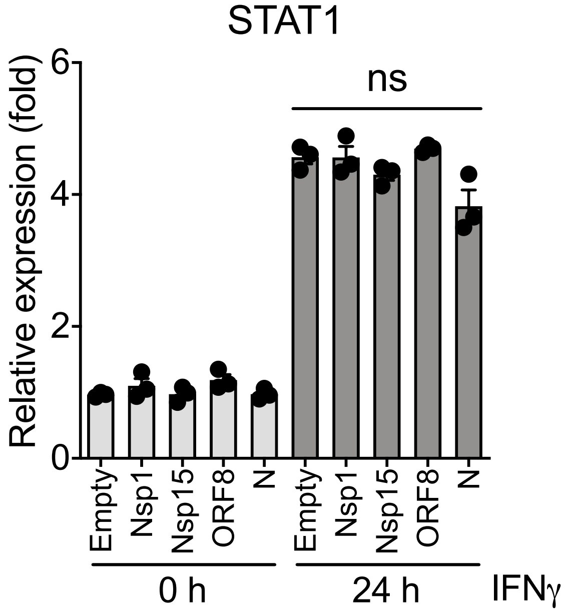

Supplementary Fig. 7. SARS-CoV-2 Nsp1, Nsp15, ORF8, and N do not affect IFNγ-induced STAT1 expression Quantitative real-time PCR analysis of STAT1 gene expression level in the indicated SARS-CoV-2 gene expressing HEK293T cells stimulated with IFNγ (100 U/ml) for the indicated time. The results are from 3 independent experiments. Two-tailed unpaired *t*-test, ns, not significant. The error bars represent mean values ± SD.

Supplementary Fig. 8.

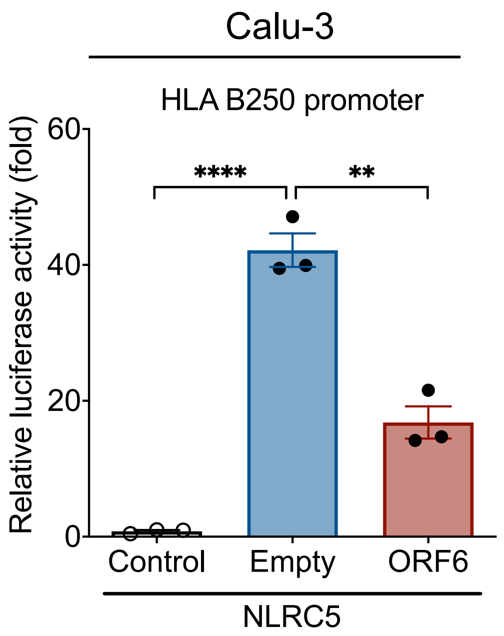

Supplementary Fig. 8. SARS-CoV-2 ORF6 inhibits NLRC5 CITA function in lung epithelial carcinoma cells Effect of SARS-CoV-2 ORF6 on NLRC5-mediated HLA-B promoter activity in Calu-3 cells. Calu-3 cells were transfected with empty control or HLA-B proximal promoter reporter construct, and plasmids expressing NLRC5 along with an empty or with SARS-CoV-2 ORF6 expressing plasmids. At 36 h after transfection, cells were collected to measure the luciferase activity. The results are from 3 independent experiments. Two-tailed unpaired *t*-test, **, *P*<0.01; ****, *P*<0.0001. The error bars depict mean values ± SD. The exact *p*-values are available in the Source Data.

Supplementary Fig. 9.

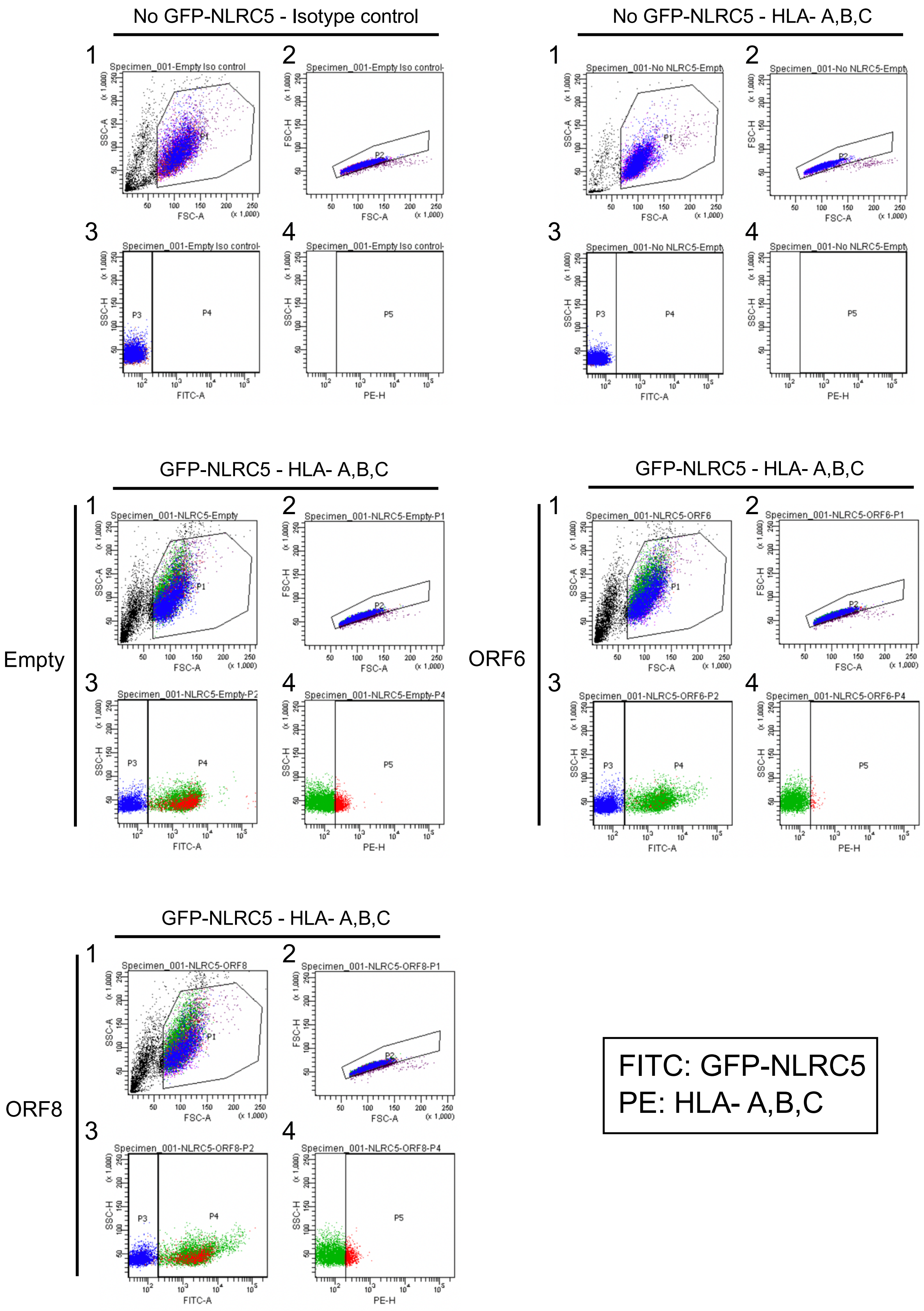

Supplementary Fig. 9. SARS-CoV-2 ORF6 inhibits NLRC5-mediated MHC class I surface expression The gating strategy for detection of the HLA- A,B,C surface expression corresponding to Fig. 5c. The gating orders are indicated by numbers. Both isotype control and anti-HLA- A,B,C antibodies are conjugated with PE. The FITC signal was used for gating the GFP-NLRC5 population.

Supplementary Fig. 10.

a

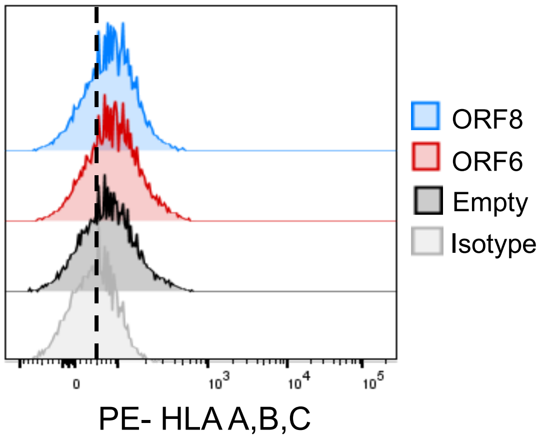

b

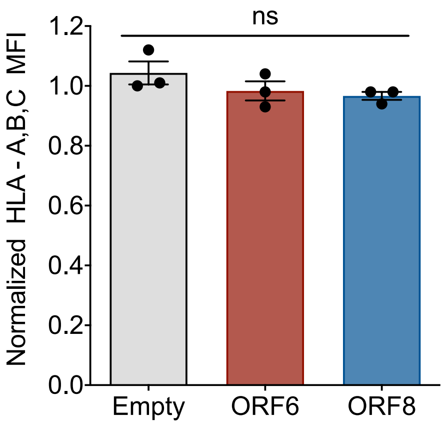

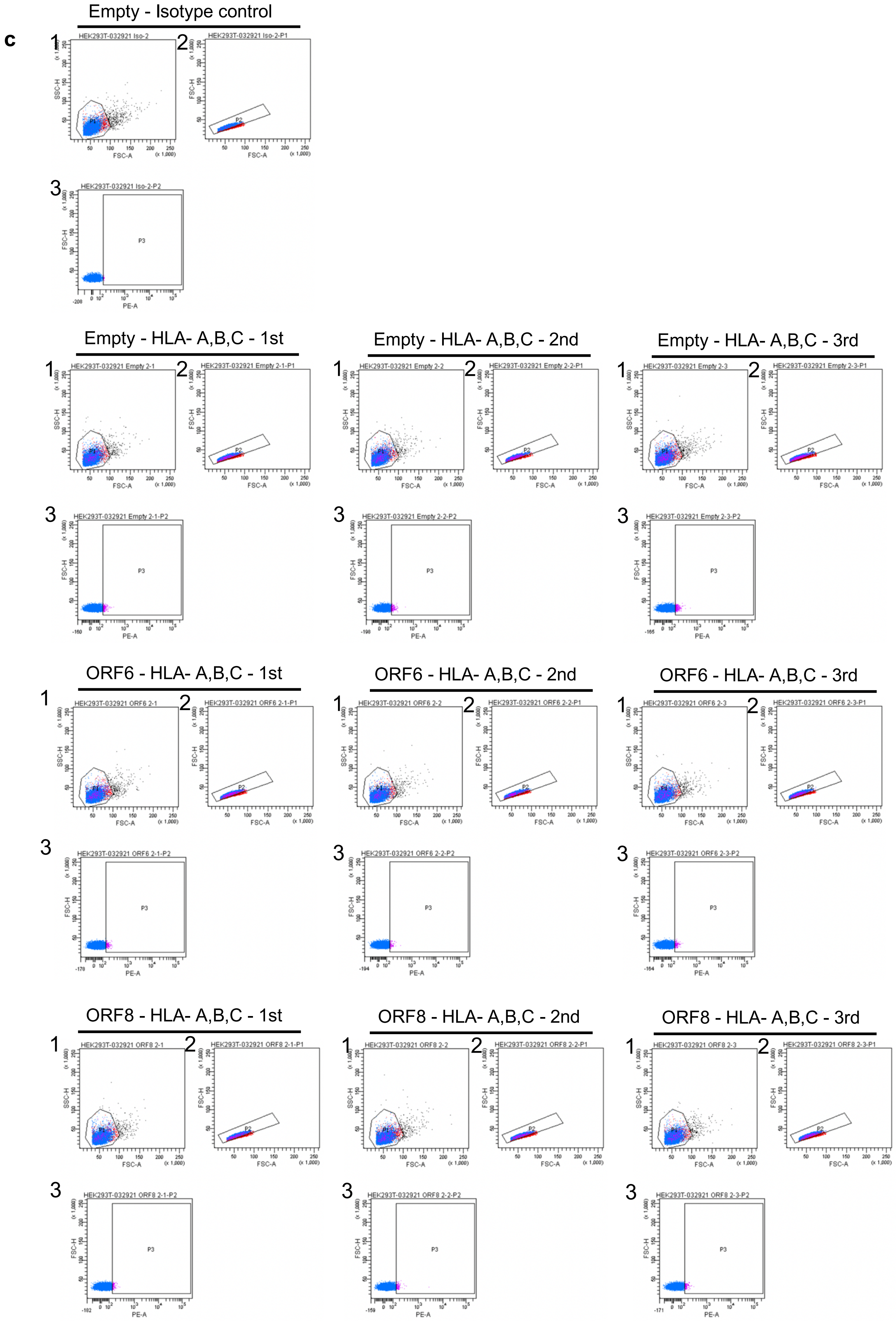
c
Empty - Isotype control
HEK293T-032921 Iso-2
HEK293T-032921 Iso-2-P1
HEK293T-032921 Iso-2-P2
Empty - HLA- A,B,C - 1st
Empty - HLA- A,B,C - 2nd
Empty - HLA- A,B,C - 3rd
HEK293T-032921 Empty 2-1
HEK293T-032921 Empty 2-1-P1
HEK293T-032921 Empty 2-2
HEK293T-032921 Empty 2-2-P1
HEK293T-032921 Empty 2-3
HEK293T-032921 Empty 2-3-P1
HEK293T-032921 Empty 2-1-P2
HEK293T-032921 Empty 2-2-P2
HEK293T-032921 Empty 2-3-P2
ORF6 - HLA- A,B,C - 1st
ORF6 - HLA- A,B,C - 2nd
ORF6 - HLA- A,B,C - 3rd
HEK293T-032921 ORF6 2-1
HEK293T-032921 ORF6 2-1-P1
HEK293T-032921 ORF6 2-2
HEK293T-032921 ORF6 2-2-P1
HEK293T-032921 ORF6 2-3
HEK293T-032921 ORF6 2-3-P1
HEK293T-032921 ORF6 2-1-P2
HEK293T-032921 ORF6 2-2-P2
HEK293T-032921 ORF6 2-3-P2
ORF8 - HLA- A,B,C - 1st
ORF8 - HLA- A,B,C - 2nd
ORF8 - HLA- A,B,C - 3rd
HEK293T-032921 ORF8 2-1
HEK293T-032921 ORF8 2-1-P1
HEK293T-032921 ORF8 2-2
HEK293T-032921 ORF8 2-2-P1
HEK293T-032921 ORF8 2-3
HEK293T-032921 ORF8 2-3-P1
HEK293T-032921 ORF8 2-1-P2
HEK293T-032921 ORF8 2-2-P2
HEK293T-032921 ORF8 2-3-P2
SSC-H
FSC-A
FSC-H
PE-A

Supplementary Fig. 10. Basal level of the MHC class I surface expression in steady-state condition is not affected by ORF6 or ORF8. The surface expression of the HLA- A, B, and C on steady-state HEK293T cells was measured by flow cytometry analysis using either isotype control or antibody targeting HLA- A, B, and C and shown with (**a**) histogram and (**b**) mean fluorescence intensity (MFI). The results shown here are from 3 independent experiments. Two-tailed unpaired *t*-test, ns, not significant. The error bars represent mean values ± SD. (**c**) Gating strategy for detection of the HLA- A,B,C surface expression. The gating orders are indicated by numbers. Both isotype control and anti-HLA- A,B,C antibodies are conjugated with PE.

Supplementary Fig. 11.

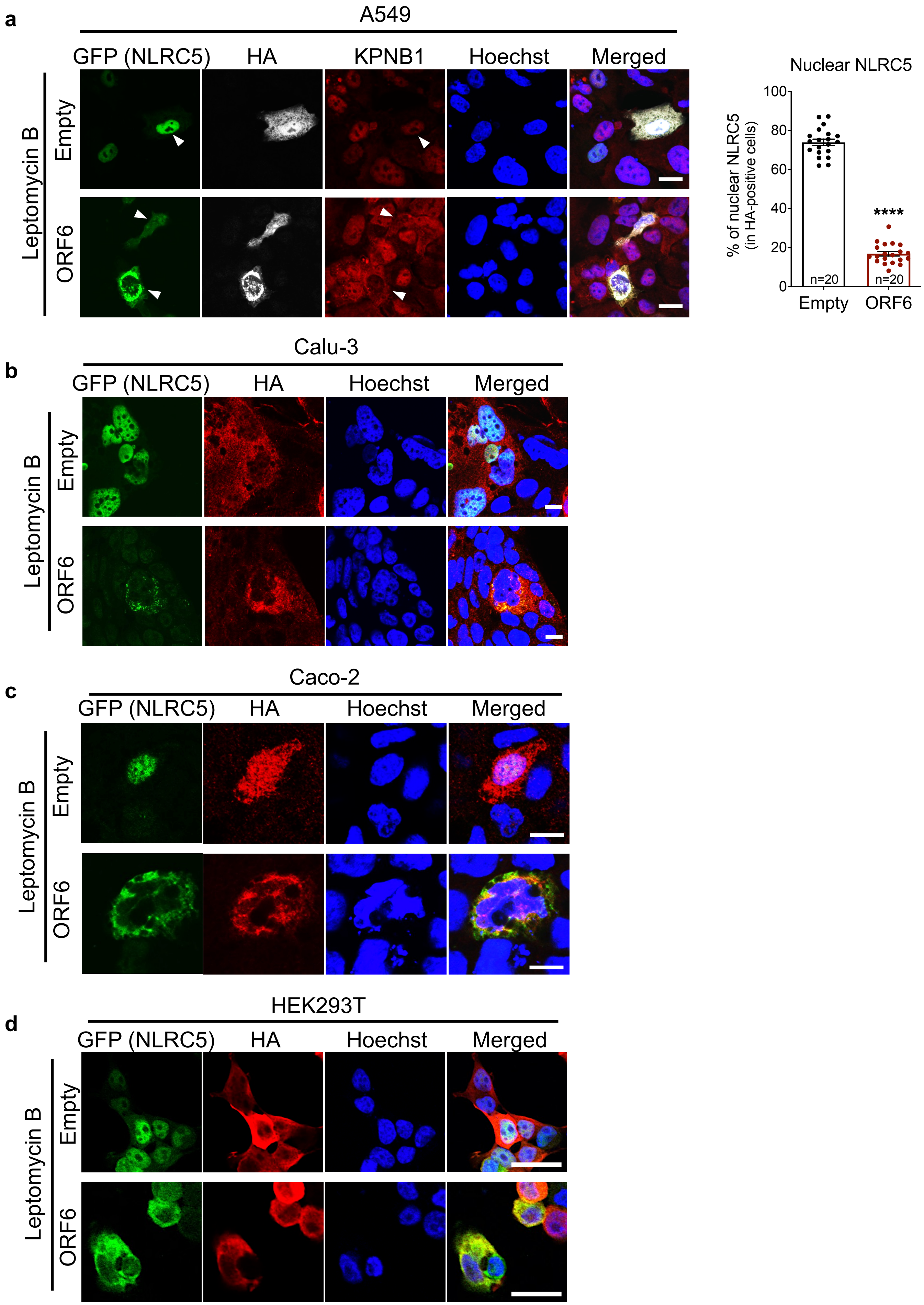

Supplementary Fig. 11. SARS-CoV-2 ORF6 inhibits nuclear translocation of NLRC5 in various epithelial cell lines Effect of SARS-CoV-2 ORF6 on the cellular localization of NLRC5. (**a**) A549, (**b**) Calu-3, (**c**) Caco-2, and (**d**) HEK293T cells were transfected with plasmids expressing GFP-tagged NLRC5, along with either HA alone or HA-tagged SARS-CoV-2 ORF6. At 24 h after transfection, cells were treated with 100 nM of Leptomycin B for 8 h, and then cellular localization of the indicated proteins was analyzed by a confocal microscope. Cellular localization of the endogenous KPNB1 in HA-empty or HA-ORF6 expressing A549 cells was indicated with a white arrow. Quantitative comparison of the nuclear NLRC5 signal intensity (% of nuclear signal intensity / total cell signal intensity) in A549 cells is shown with a bar graph analyzed by ImageJ. The scale bar indicates 20 μm. Two-tailed unpaired *t*-test, ****, $P<0.0001$. The error bars depict mean values ± SD. The results shown are the representative data from the multiple images taken (2-8 images, n=2 biological replicates) for each experiment.

Supplementary Fig. 12.

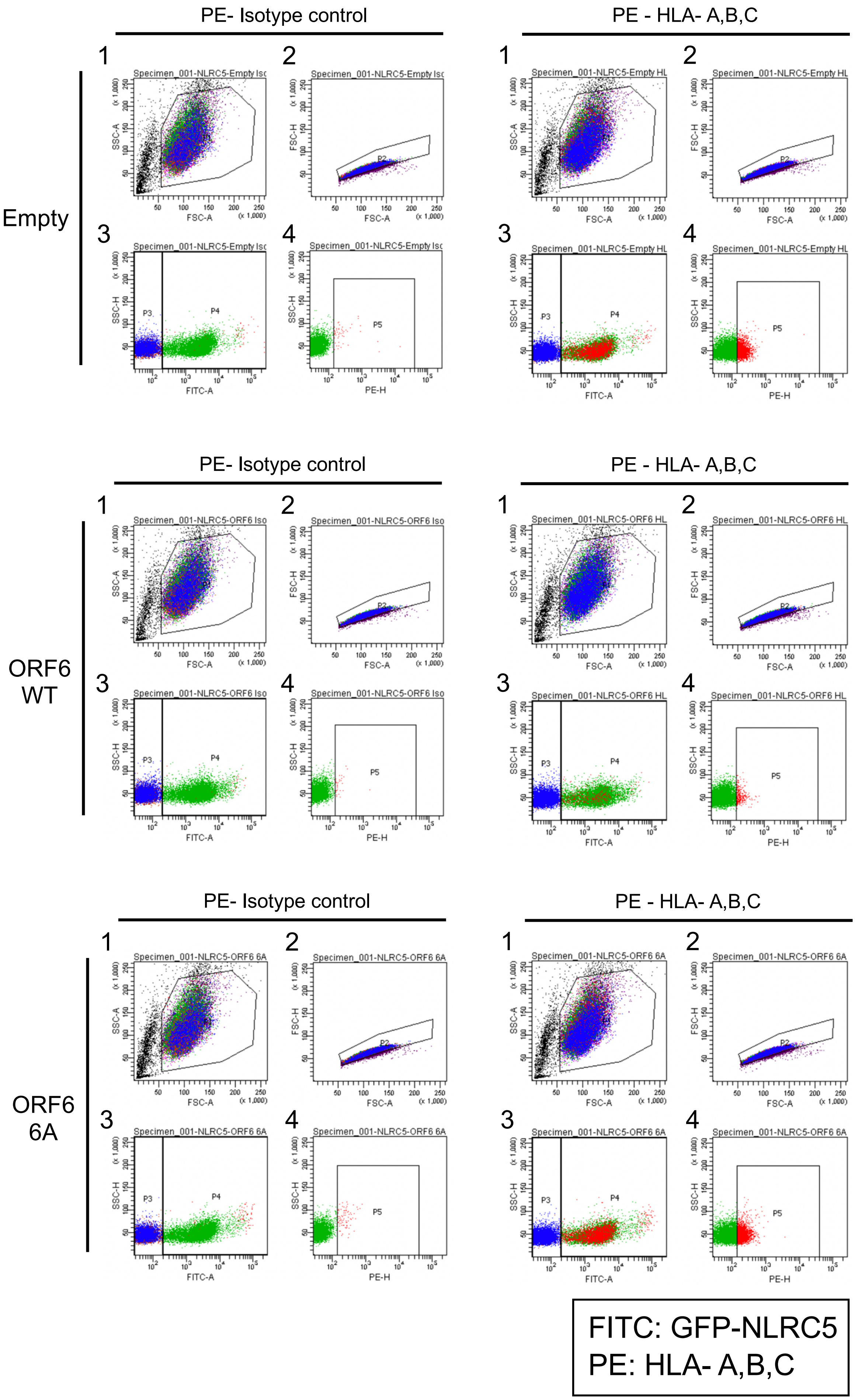

Supplementary Fig. 12. Carboxy terminus of SARS-CoV-2 ORF6 is involved in inhibition of the NLRC5-mediated MHC class I surface expression The gating strategy for detection of HLA- A,B,C surface expression corresponding to Fig. 7b. The gating orders are indicated by numbers. Both isotype control and anti-HLA- A,B,C antibodies are conjugated with PE. The FITC signal was used for gating the GFP-NLRC5 population.

Supplementary Table 1. DNA oligo used for cloning.

Use	Primer name and sequence (5' to 3')		Note
Cloning or subcloning of SARS-CoV-2 genes	nsp1-F	ATAGGATCCATGGAGAGCCTTGTC	Using BamHI and XbaI
	nsp1-R	ATTCTAGATTACTGCTATGTTTAGTGTTCC	
	nsp15-F	ATAGGATCCATGAGTTTAGAAAATGT	
	nsp15-R	ATTCTAGATTATTGTAATTTTGGGTA	
	ORF6-F	ATAGGATCCATGTTTCATCTCGTTG	
	ORF6-R	ATTCTAGATTAATCAATCTCCATTGGTTG	
	ORF8-F	ATAGGATCCATGAAATTTCTTGTTTTC	
	ORF8-R	ATTCTAGATTAGATGAAATCTAAAACAAC	
	N-F	ATAGGATCCATGTCTGATAATGGAC	
	N-R	ATTCTAGATTAGGCCTGAGTTGAG	
Cloning or subcloning of human karyopherin genes	KPNA1-F	CTAGAATTCACCACCCCAGGAAAAGAGAACTTT	Using EcoRI and XbaI
	KPNA1-R	AAGTCTAGATCAAAGCTGGAAACCTTCCATAGG	
	KPNA2-F	AATGGATCCATGTCCACCAACGAGAATGCTAATA	Using BamHI and XbaI
	KPNA2-R	AATTCTAGACTAAAAGTTAAAGGTCCCAGGAGCC	
	KPNA3-F	AAAGAATTCATGGCCGAGAACCCCAGCTTGGAGAA	Using EcoRI and XhoI
	KPNA3-R	CGGCTCGAGTTAAAAATTAAATTCTTTTGTTTGAAGG TTGGC	
	KPNA4-F	AAAGGATCCATGGCGGACAACGAGAAACTGGACA	Using BamHI and XhoI
	KPNA4-R	GGACTCGAGCTAAAACTGGAACCCTTCTGTTGGT	
	KPNA5-F	AAAGAATTCATGGATGCCATGGCTAGTCCAGGGAAA	Using EcoRI and XhoI
	KPNA5-R	GGACTCGAGTTAAAGTTGAAATCCATCCATTGGTGC	
	KPNA6-F	AAAGAATTCGAGACCATGGCGAGCCCAGGGAAAGA CAA	Using EcoRI and XbaI
	KPNA6-R	AAATCTAGATTATAGCTGGAAGCCCTCCATGGGGGC CT	
	KPNB1 WT-F	AAAGGATCCATGGAGCTGATCACCATTCTCGAG	Using BamHI and XbaI
	KPNB1 WT-R	GCCTCTAGATCAAGCTTGGTTCTTCAGTTTCCT	
	KPNB1 DN-F	AAAGGATCCATGGCAAATCCAGGAAACAGTCAGGTT	
	KPNB1 DN-R	AGGTCTAGATTAAGCACTGAGACCCTCAATCAGACA	
Cloning of the reporter gene for IRF1 GAS element	IRF1 3 × GAS-F	CCTGATTTCCCCGAAATGACCTGATTTCCCCGAAAT GACCTGATTTCCCCGAAATGACC	Using KpnI and XhoI (Synthesized oligo)
	IRF1 3 × GAS-R	TCGAGGTCATTTCGGGGAAATCAGGTCATTTCGGGG AAATCAGGTCATTTCGGGGAAATCAGGGTAC	
Cloning of the reporter gene for human NLRC5 promoter	NLRC5 promoter-F	GGGGTACCCTGGGAGTCACACAC	Using KpnI and XhoI
	NLRC5 promoter-R	CCCTCGAGCTCCGTGCAGAATTCGA	

Supplementary Table 2. DNA oligo used for qPCR analysis.

Use	Oligo name and sequence (5' to 3')		Note
qPCR primers for human gene analysis	NLRC5-F	GTCATCCGCCTCTGGAATAAC	SYBR green system
	NLRC5-R	CTGGTTGTCAAAGAAGGCAAAG	
	HLA A-F	AAAAGGAGGGAGTTACACTCAGG	
	HLA A-R	GCTGTGAGGGACACATCAGAG	
	HLA B-F	CTACCCTGCGGAGATCA	
	HLA B-R	ACAGCCCAGGCCAGCAACA	
	B2M-F	CCACTGAAAAAGATGAGTATGCCT	
	B2M-R	CCAATCCAAATGCGGCATCTTCA	
	TAP1-F	AGGGCTGGCTGGCTGCTTTGA	
	TAP1-R	ACGTGGCCCATGGTGTTGTTAT	
	PSMB9-F	CGAGAGGACTTGTCTGCACATC	
	PSMB9-R	CACCAATGGCAAAAGGCTGTCG	
	STAT1-F	ATGGCAGTCTGGCGGCTGAATT	
	STAT1-R	CCAAACCAGGCTGGCACAATTG	
	IRF1-F	GAGGAGGTGAAAGACCAGAGCA	
	IRF1-R	TAGCATCTCGGCTGGACTTCGA	
	IFNB-F	AGCTGCAGCAGTTCCAGAAG	
	IFNB-R	AGTCTCATTCCAGCCAGTGC	
	IL6-F	AGACAGCCACTCACCTCTTCAG	
	IL6-R	TTCTGCCAGTGCCTCTTTGCTG	
	TNF-F	CTCTTCTGCCTGCTGCACTTTG	
	TNF-R	ATGGGCTACAGGCTTGTCACTC	
	ISG15-F	AGATCACCCAGAAGATCG	
	ISG15-R	TGTTATTCCTCACCAGGATG	
	OAS2-F	GCTTCCGACAATCAACAGCCAAG	
	OAS2-R	CTTGACGATTTTGTGCCGCTCG	
	IFI6-F	TGATGAGCTGGTCTGCGATCCT	
	IFI6-R	GTAGCCCATCAGGGCACCAATA	
	GAPDH-F	GAAGGTGAAGGTCGGAGT	
	GAPDH-R	GAAGATGGTGATGGGATTTC	
qPCR primers for SARS-CoV-2 gene analysis	SARS-CoV-2-E-F	ACAGGTACGTTAATAGTTAATAGCGT	
	SARS-CoV-2-E-R	ATATTGCAGCAGTACGCACACA	
