# Supplementary material for: SARS-CoV-2 inhibits induction of the MHC class I pathway by targeting the STAT1-IRF1-NLRC5 axis
Source: Nat Commun. 2021 Nov 15;12:6602. doi: 10.1038/s41467-021-26910-8 (PMC8594428; doi:10.1038/s41467-021-26910-8)
Supplement: Supplementary file 3 — Reporting summary [file 41467_2021_26910_MOESM3_ESM.pdf]

nature research

Corresponding author(s): Koichi S. Kobayashi

Last updated by author(s): May 7, 2021

Reporting Summary

Nature Research wishes to improve the reproducibility of the work that we publish. This form provides structure for consistency and transparency in reporting. For further information on Nature Research policies, see our Editorial Policies and the Editorial Policy Checklist.

Statistics

For all statistical analyses, confirm that the following items are present in the figure legend, table legend, main text, or Methods section.

n/a	Confirmed	
☐	☒	The exact sample size (*n*) for each experimental group/condition, given as a discrete number and unit of measurement
☐	☒	A statement on whether measurements were taken from distinct samples or whether the same sample was measured repeatedly
☐	☒	The statistical test(s) used AND whether they are one- or two-sided<br>*Only common tests should be described solely by name; describe more complex techniques in the Methods section.*
☐	☒	A description of all covariates tested
☐	☒	A description of any assumptions or corrections, such as tests of normality and adjustment for multiple comparisons
☐	☒	A full description of the statistical parameters including central tendency (e.g. means) or other basic estimates (e.g. regression coefficient) AND variation (e.g. standard deviation) or associated estimates of uncertainty (e.g. confidence intervals)
☐	☒	For null hypothesis testing, the test statistic (e.g. *F*, *t*, *r*) with confidence intervals, effect sizes, degrees of freedom and *P* value noted<br>*Give P values as exact values whenever suitable.*
☒	☐	For Bayesian analysis, information on the choice of priors and Markov chain Monte Carlo settings
☒	☐	For hierarchical and complex designs, identification of the appropriate level for tests and full reporting of outcomes
☒	☐	Estimates of effect sizes (e.g. Cohen's *d*, Pearson's *r*), indicating how they were calculated

Our web collection on statistics for biologists contains articles on many of the points above.

Software and code

Policy information about availability of computer code

Data collection	No software was used for data collection
Data analysis	For bioinformatic analyses (Figure 1, Supplementary Figure 1a), R version 3.6.0 was used with RStudio 1.1.463 and the following libraries: ggpubr 0.2.5, dplyr 0.8.4, DESeq2 1.26, ComplexHeatmap 2.2.0, stringr 1.4.0, ggplot2 3.3.2, dplyr 1.0.7, plyr 1.8.4 and ggbeeswarm 0.6.0. Scripts used to perform processing and generate figures can be found at https://github.com/scho75/RNA-seq-data-mining-analysis.<br>All non-RNA seq statistical analyses were performed as indicated in figure legends using GraphPad Prism 9 (GraphPad Software, USA).<br>For quantification of the immunofluorescence images and protein bands from Western blot analysis was performed with ImageJ software.

For manuscripts utilizing custom algorithms or software that are central to the research but not yet described in published literature, software must be made available to editors and reviewers. We strongly encourage code deposition in a community repository (e.g. GitHub). See the Nature Research guidelines for submitting code & software for further information.

Data

Policy information about availability of data

All manuscripts must include a data availability statement. This statement should provide the following information, where applicable:

- Accession codes, unique identifiers, or web links for publicly available datasets
- A list of figures that have associated raw data
- A description of any restrictions on data availability

Data availability
The RNAseq data used in this study are available in the NCBI Gene Expression Omnibus (GEO) Repository under accession code GSE152075 [https://www.ncbi.nlm.nih.gov/geo/query/acc.cgi?acc=GSE152075] for the nasopharyngeal swab dataset and under accession code GSE147507 [https://www.ncbi.nlm.nih.gov/geo/query/acc.cgi?acc=GSE147507] for the normal human bronchial epithelial cell dataset. Source data for scRNA-seq data in Supplementary

Figure 1b can be accessed on the cellxgene platform (https://cellxgene.cziscience.com/e/krasnow_lab_human_lung_cell_atlas_10x-1-remixed.cxg/). The source data underlying Figs 1d, 2a-b, 3a-f, 4a-e, 5a-b, 5d-e, 6b-f, 7a, 7c-d and Supplementary Figures 2, 3a-b, 6a-b, 7, 8, 10b, and 11a are provided as a Source Data file. No additional dataset has been generated in the current study.

Code availability
Scripts used to perform processing and generate figures can be found at https://github.com/scho75/RNA-seq-data-mining-analysis.

Field-specific reporting

Please select the one below that is the best fit for your research. If you are not sure, read the appropriate sections before making your selection.

- [x] Life sciences
- [ ] Behavioural & social sciences
- [ ] Ecological, evolutionary & environmental sciences

For a reference copy of the document with all sections, see nature.com/documents/nr-reporting-summary-flat.pdf

Life sciences study design

All studies must disclose on these points even when the disclosure is negative.

Sample size	Sample sizes were not predetermined based on statistical methods, but were chosen according to the standards of the field (at least three independent biological replicates for each condition), which generated a sufficient statistics for the effect sizes of interest.
Data exclusions	No data were excluded.
Replication	All studies performed by RT-qPCR or reporter assay were conducted with three independent experiments and the p-value of each experiment is provided by Student's t-test. For immunofluorescence analysis, 16 to 60 cells per group were counted and represented as a bar graph with a p-value by Student's t-test. For the quantification of the Western blot band shown in Fig. 5e, three independent experiments were conducted and the band intensity was quantified using ImageJ software. The average band intensity was shown with a bar graph.
Randomization	No randomization was necessary for this study because investigators were comparing designed samples under well controlled conditions (e.g. mock vs infected or Empty vector control vs effector protein encoding vector). No human or animal subjects were used in the study. Randomization is not generally used in this field.
Blinding	Investigators were not blinded. Blinding during collection was not needed because conditions were well controlled. Blinding is also not necessary because the results are quantitative and did not require subjective judgment or interpretation. Blinding is not typically used in the field.

Reporting for specific materials, systems and methods

We require information from authors about some types of materials, experimental systems and methods used in many studies. Here, indicate whether each material, system or method listed is relevant to your study. If you are not sure if a list item applies to your research, read the appropriate section before selecting a response.

Materials & experimental systems

n/a	Involved in the study	
[ ]	[x]	Antibodies
[ ]	[x]	Eukaryotic cell lines
[x]	[ ]	Palaeontology and archaeology
[x]	[ ]	Animals and other organisms
[x]	[ ]	Human research participants
[x]	[ ]	Clinical data
[x]	[ ]	Dual use research of concern

Methods

n/a	Involved in the study	
[x]	[ ]	ChIP-seq
[ ]	[x]	Flow cytometry
[x]	[ ]	MRI-based neuroimaging

Antibodies

Antibodies used

anti-FLAG antibody (Sigma), Clone: M2, Cat#: F3165-1MG
anti-HA antibody (BioLegend), Clone: 16B12, Cat#: 901513
anti-TBK1 antibody (Cell Signaling), Clone: E8I3G, Cat#: 38066
anti-phospho-TBK1 antibody (Cell Signaling), Clone: D52C2, Cat#: 5483
anti-STAT1 antibody (Cell Signaling), Clone: D1K9Y, Cat#: 14994
anti-phospho-STAT1 antibody (Cell Signaling), Clone: 58D6, Cat#: 9167
anti-SARS-CoV-2 N antibody (GeneTex), Clone: polyclonal, Cat#: GTX135357
anti-GFP antibody (Proteintech), Clone: 1E10H7, Cat#: 66002-1-Ig
anti-Lamin B1 (Cell Signaling), Clone: D9V6H, Cat#: 13435
anti-Tubulin (Proteintech), Clone: polyclonal, Cat#: 11224-1-AP

	anti-GAPDH (Proteintech), Clone: 1E6D9, Cat#: 60004-1-Ig<br>anti-beta actin (Proteintech), Clone: 2D4H5, Cat#: 66009-1-Ig<br>anti-rabbit IgG with HRP conjugate (GE Healthcare), Cat#: NA934V<br>anti-mouse IgG with HRP conjugate (GE Healthcare), Cat#: NA931V<br>anti-human HLA-A, B, C antibody, PE-conjugated (BioLegend), Clone: W6/32, Cat#: 311406<br>PE-conjugated isotype control, mouse IgG2a, k (BioLegend), Clone: MOPC-173, Cat#: 400213
Validation	Antibodies were chosen based on the validation statements for species (Human) and application (Western blot, Immunofluorescence or Flow Cytometry) on the manufacturer's website.

Eukaryotic cell lines

Policy information about cell lines

Cell line source(s)	Calu-3, ATCC , HTB-55™<br>HeLa, ATCC, CCL-2™<br>HEK293T, ATCC, CRL-11268™<br>A549, RIKEN Bio BRC, RCB0098<br>Caco-2, RIKEN Bio BRC, RCB0988<br>Huh-7, RIKEN Bio BRC, RCB1366
Authentication	We have obtained low-passage cell lines from the providers and all cell lines are authenticated by STR method.
Mycoplasma contamination	All cell lines were negative for mycoplasma contamination.
Commonly misidentified lines<br>(See ICLAC register)	None of the used cell lines is listed in ICLAC database.

Flow Cytometry

Plots

Confirm that:

- [x] The axis labels state the marker and fluorochrome used (e.g. CD4-FITC).
- [x] The axis scales are clearly visible. Include numbers along axes only for bottom left plot of group (a 'group' is an analysis of identical markers).
- [x] All plots are contour plots with outliers or pseudocolor plots.
- [x] A numerical value for number of cells or percentage (with statistics) is provided.

Methodology

Sample preparation	For sample preparation, cell cultures were harvested by trypsinization. Initial culture medium and wash buffer were collected to include detached cells. Cells were centrifuged (250g, 5min) and resuspended in the culture media. Then cell number was determined by a hemocytometer. After centrifugation, indicated numbers of cells were seeded in the culture plate.<br>On the next day, cell cultures were treated as indicated in the manuscript and harvested by a cell scraper. Cells were centrifuged (250g, 5min) and resuspended in staining buffer (2% BSA in PBS). For the analysis of HLA surface expression, cells were stained with either isotype control (PE-conjugated mouse IgG2a, k (BioLegend), Clone: MOPC-173, Cat#: 400213, 1:100 dilution) or anti-human HLA- A,B,C antibody (PE-conjugated (BioLegend), Clone: W6/32, Cat#: 311406, 1:100 dilution) on ice for 30 min. After staining, cells were washed three times with staining buffer. Then, samples were analyzed by flow cytometry using BD Canto (BD Biosciences) at least 10.000 events were acquired per sample.
Instrument	BD FACS Canto (BD Biosciences) equipped with 405nm,488nm and 640nm lasers.
Software	FlowJo
Cell population abundance	Cell sorting not employed
Gating strategy	Using the FSC/SSC gating, debris was removed by gating on the main cell population. For choosing a single cell population, FSC-H/FSC-A gating was used. The background intensity was determined by isotype control antibody. The positivity threshold for each cell line was defined based on the unstimulated sample (negative control). The identical positivity threshold was then applied to all samples within each experiment.

- [x] Tick this box to confirm that a figure exemplifying the gating strategy is provided in the Supplementary Information.
